# Evaluation of Drug Susceptibility of Microorganisms in Odontogenic Inflammations and Dental Surgery Procedures Performed on an Outpatient Basis

**DOI:** 10.1155/2019/2010453

**Published:** 2019-10-07

**Authors:** Mateusz Bogacz, Tadeusz Morawiec, Joanna Śmieszek-Wilczewska, Katarzyna Janowska-Bogacz, Anna Bubiłek-Bogacz, Rafał Rój, Karolina Pinocy, Anna Mertas

**Affiliations:** ^1^Department of Oral Surgery, School of Medicine with the Division of Dentistry in Zabrze, Medical University of Silesia in Katowice, Pl. Akademicki 17, Bytom 41-902, Poland; ^2^Department of Conservative Dentistry with Endodontics, School of Medicine with the Division of Dentistry in Zabrze, Medical University of Silesia in Katowice, Plac Akademicki 17, Bytom 41-902, Poland; ^3^Department of Prosthetic Dentistry, School of Medicine with the Division of Dentistry in Zabrze, Medical University of Silesia in Katowice, Plac Akademicki 17, Bytom 41-902, Poland; ^4^Department of Microbiology and Immunology, School of Medicine with the Division of Dentistry in Zabrze, Medical University of Silesia in Katowice, Jordana 19, Zabrze 41-808, Poland

## Abstract

Bacterial infections are the most common cause of purulent soft tissue inflammations in the head and neck area. These bacteria are also responsible for the majority of inflammatory complications after third molar removal. The key to success of antibacterial treatment in both cases is the use of an appropriate antibacterial agent. The aim of the study was to evaluate the susceptibility profile of bacteria isolated from material collected from patients with intraoral odontogenic abscesses. The test material consisted of swabs taken from the odontogenic abscesses, after their incision and drainage. Another swab was collected from the lesion area, 10 days after the initial visit. Results were compared with an identical study conducted on a control group of healthy patients, who had undergone third molar removal. Bacteria identified in this study consisted of aerobic and anaerobic strains, both Gram-positive and Gram-negative. According to the EUCAST guidelines, none of the tested antibiotics was recommended for all identified bacteria. The percentage of bacterial strains sensitive to amoxicillin and clavulanic acid was 78.13% and 81.48% in the study and control groups, respectively, whereas, the percentage of those sensitive to clindamycin was 96.43% and 80.00%, respectively. For Gram-negative aerobic bacteria, gentamicin and ciprofloxacin were among medications affecting all cultured species. 100.00% of strains were found to be susceptible to these antibiotics. Statistically significant relationship between the presence of Gram-negative aerobic strains and the occurrence of complications was found. In the case of the most frequently occurring bacteria in the study, amoxicillin with clavulanic acid and clindamycin were shown to be very effective. In cases of severe purulent odontogenic inflammations, it is recommended to use a combination of antibiotics. Amoxicillin with ciprofloxacin and clindamycin with cefuroxime seem to be the proper choices based on the results of this study.

## 1. Introduction

Bacterial infections are the most common cause of purulent soft tissue inflammations in the head and neck area [1]. Their occurrence is favoured by a large variety of oral microbiota and lesions of dental tissues and the periodontium [2, 3]. The causes of infections are divided into odontogenic and nonodontogenic, with 70–90% of cases belonging to the first group. The most common odontogenic causes are gangrenous teeth, complicated third molar eruption, infected dental cysts, residual tooth roots, and complications after endodontic treatment [4–6]. The most frequent cause of the development of periapical inflammatory changes is pulpitis, which results from negligence in conservative treatment [7, 8]. The bacterial antigens present in the inflamed pulp tissue stimulate the specific and nonspecific immune responses of the body, but it is usually not possible to completely eradicate the infection [9]. A chronic inflammatory change develops in the periapical region of the infected tooth. The central part of the lesion exhibits the largest accumulation of neutrophilic granulocytes, forming foci of colliquative necrosis, in which purulent exudate accumulates. Distribution of periodontal collagen fibres causes merging of smaller purulent foci, which ultimately leads to the formation of a periapical abscess [10]. Chronic inflammations are usually asymptomatic and almost always lead to bone resorption around the tooth root, giving characteristic lucencies in the X-ray image. This is not the case with acute inflammations, the course of which is most often associated with severe pain and swelling of soft tissues. Acute inflammations do not show any characteristic features in the X-ray image. In some cases, they can manifest themselves in the widening of the periodontal ligament space [11]. However, the probable causes of such inflammations are often visible—most often deep carious lesions, extensive fillings, including the pulp chamber or in the close vicinity of it. Acute inflammation can be both a primary condition and exacerbation of chronic inflammation. It is characterized by a fast course, during which there is no natural barrier to the spread of infection. This type of inflammation is considered more dangerous, as microorganisms penetrating the periapical tissue can spread to other parts of the head and neck [9, 11, 12]. Clinically, we distinguish different forms of odontogenic inflammation, which, depending on the severity of the disease, differ significantly in terms of symptoms reported by the patient and their treatment. Root canal treatment is recommended for the management of periapical inflammations and abscesses. In the case of purulent soft tissue inflammation, it is necessary to perform intra- or extraoral incisions to obtain effective drainage. Seton placed inside the abscess is replaced daily until the complete evacuation of the purulent exudate, and the patient attends checkups until the clinical condition is significantly improved [13–15]. In the case of oral inflammation with symptoms that might be life-threatening, the treatment should be complemented by empiric antibiotic therapy. The microbiome of the oral cavity consists of aerobic and anaerobic species, both Gram-positive and Gram-negative; therefore, no antibiotic is effective against all of them. The key to success in empirical antibiotic therapy is to identify those bacteria species that are most frequently related to ongoing infection and assess their susceptibility to drugs that can be used efficiently in everyday dental practice. Antibiotics are often used in dentistry before planned surgery in order to minimize the risk of postoperative infections. This procedure is frequently employed in the impacted third molar (ITM) surgery. Compared to simple tooth extraction, the ITM surgery has a greater risk of penetration of microorganisms present in the oral cavity into the tissues. Such a condition can impair wound healing; in some situations, it can also lead to the development of a generalized infection. This is particularly dangerous for patients with systemic diseases and hence an already weakened immune system. In order to ensure proper healing, patients need to maintain proper oral hygiene (including removal of dental plaque) prior to the treatment. It is also necessary to instruct the patient on the principles of oral hygiene. The patient compliance with the recommendations should be checked according to appropriate indicators, including the Approximal Plaque Index (API). Patients who are eligible for elective ITM surgery should not have clinical symptoms of an ongoing inflammatory process. Otherwise, it is necessary to precede the surgical procedure with an appropriate conservative treatment.

## 2. Materials and Methods

### 2.1. Study and Control Groups and Patient Examination

The study included 52 patients, who were divided into two groups:

1. Study group (26 patients)—patients who were diagnosed with the following:
  - Submucous abscess, requiring removal of causative teeth, as well as soft tissue incision and setoning—2 women and 13 men
  - Periapical abscess, requiring only removal of causative teeth (purulent exudate drained through the alveolus)—3 women and 8 men
2. Control group (26 patients, including 19 women and 7 men)—patients without acute inflammation, referred for a planned ITM surgery

**Figure 1:**
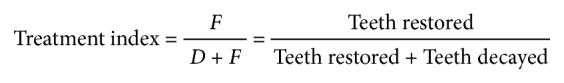
Calculating Treatment Index based on the *F* and *D* scores from the DMF Index.

A dental diagram was made during the study, based on which the DMF (Decay-Missing-Filled) Index was calculated by counting the number of decayed (*D*), missing (*M*) due to caries, and restored (filled—*F*) teeth in each patient. Treatment Index was also calculated for each patient (Figure 1).

The Approximal Plaque Index (API) was also calculated at each visit. Before the procedure, patients in both groups underwent a radiological examination in the form of point, panoramic, and, if necessary, volumetric tomography. The examinations included the general state of health of the patients, medications they were taking, and the current treatment of the existing inflammation. The first stage of treatment in patients from the study group diagnosed with submucous, subperiosteal, or subcutaneous abscess involved incision of the purulent lesion to evacuate its content. Due to contraindications to endodontic treatment (poor oral hygiene and extensive tooth crown damage), the next stage involved removal of the causative tooth. In the case of periapical abscesses, the surgical part of the treatment consisted only of removing the causative tooth. The next stage of the procedure was collection of swabs for microbiological examination. Material collected for testing was purulent exudate from inflammatory foci in oral tissues. Directly prior to the collection, the lesion area was isolated with sterile gauze and disinfected. The first portion of purulent content was removed, and then a smear swab was taken from the deepest possible site (using the sterile swab) (study I). In cases requiring extraction of the causative tooth with no indications for incision, the place of swab collection was the deepest possible spot of tooth alveolus, after removal of the first portion of purulent exudate. The swabs were placed in a transport medium for aerobic and anaerobic microorganisms. In accordance with routine procedures, patients were required to report for control visits every day over the next several days to assess the healing of the lesion. At each visit, the general and local condition of the patient was assessed and the seton was changed. Healing of these types of lesions usually takes 7 to 10 days. The last visit was set for 10 days after the beginning of the treatment, and a control swab from the area of the lesion was collected (study II).

The control group consisted of patients with a planned ITM surgery. On the day of the surgery, a clinical and radiological examination was carried out (as described above), followed by collection of a swab for microbiological examination from the surgical area (study I). The next step was to perform the ITM surgery, and the procedure was carried out as follows:

1. Incision and detachment of the mucoperiosteal flap
2. Exposition of the impacted tooth and its separation with a burr
3. Removal of the tooth along with the surrounding pathological lesions (follicular cysts, tooth follicle and granulomatous lesions)
4. Wound management with sutures and pressure dressing

The control visit was set for the 10^th^ day after the procedure. After evaluating the healing of the wound, a swab was taken for microbiological tests from the surgical area (study II), and then sutures were removed under topical anaesthesia.

### 2.2. Antibiotic Therapy—Indications

In the study group, every patient diagnosed with oral inflammation accompanied by possibly life-threatening symptoms (rapidly growing face swelling, trismus, significant enlargement and painfulness of the surrounding lymph nodes, impaired swallowing, and breathing), systemic symptoms such as tachycardia (with pulse over 100 bpm), or increased body temperature underwent treatment complemented by an empiric antibiotic therapy.

In the control group, every patient in whose case the ITM surgery was associated with disruption of the bone tissue continuity and every patient for whom the surgery took more than 30 minutes also underwent treatment complemented by an empiric antibiotic therapy.

In the case of indications for the implementation of antibiotic therapy, one of the following two antibiotics was used:

1. Amoxicillin (875 mg) with clavulanic acid (125 mg), 1 tablet every 12 hours for 6 days
2. Clindamycin (600 mg), 1 tablet every 12 hours for 6 days

In the present study, indications for the initiation of antibiotic therapy were found in 19 out of 26 patients (73.08%) in the study group and in 21 out of 26 (80.77%) patients in the control group.

### 2.3. Microorganism Identification and Evaluation of Drug Susceptibility

Material collected from the patients was delivered to the Microbiological Laboratory of the Chair and Department of Microbiology and Immunology in Zabrze, Medical University of Silesia in Katowice, where microbiological tests were carried out. The time from material collection until delivery to the laboratory did not exceed 2 hours. Microbiological tests were carried out using classic methods used in microbiological diagnostics. The material was seeded on appropriate culture media to amplify and isolate pure microbial cultures. Aerobic bacteria were grown on solid Columbia agar with 5% sheep blood at 37°C. Anaerobic bacteria were grown on a solid Schaedler K3 with 5% sheep blood at 37°C under anaerobic conditions obtained with the use of GENbag anaer kits (Biomerieux, Marcy-l'Etoile, France). After isolation and multiplication of cultivated microbial strains, species identification was performed using the following reagent kits (Erba-Lachema, Brno, Czech Republic): ENTEROtest 24 N, NEFERMtest 24 N, STREPTOtest 24, STAPHYtest 24, ANAEROtest 23, OXItest, PYRAtest, as well as Erba-Lachema's TNW Lite 6.5 software (Brno, Czech Republic). The following biochemical tests were also used (Biomerieux, Marcy-l'Etoile, France): Katalaza and Slidex Staph Kit. The performance, reading, and interpretation of test results were carried out in accordance with the recommendations of manufacturers of diagnostic reagent kits.

Bacterial drug susceptibility was determined using the Kirby–Bauer disk diffusion method [16] and Etest method. The implementation of this stage of the study and the interpretation of the obtained results were in accordance with the current EUCAST (European Committee on Antibiotic Susceptibility Testing) recommendations [17]. Twelve antibiotics belonging to following different classes were used in the form of discs (Oxoid Limited, Basingstoke, UK) and/or Etests (Biomerieux, Marcy-l'Etoile, France): (a) penicillins: benzylpenicillin 1 unit (P), amoxicillin with clavulanic acid 20–10 *μ*g (AUG), piperacillin with tazobactam 30–6 *μ*g (TZP), and ampicillin 10 *μ*g for Enterobacterales or 2 *μ*g for the other bacterial species (AM); (b) cephalosporins: cefuroxime 30 *μ*g, the 2^nd^ generation (CXM), and cefepime 30 *μ*g, the 4^th^ generation (FEP); (c) fluoroquinolone: ciprofloxacin 5 *μ*g (CIP); (d) aminoglycoside: gentamicin 10 *μ*g (G); (e) glycopeptide: vancomycin 5 *μ*g (Va); (f) lincosamide: clindamycin 2 *μ*g (CC); (g) nitroimidazole: metronidazole—only Etest (MZ); and (h) aminopyrimidine with sulphonamide: trimethoprim-sulfamethoxazole 1,25–23,75 *μ*g (biseptol—Bs).

Statistical analyses were carried out using the Statistica PL v. 13 software (Statsoft, Kraków, Poland), assuming the level of significance at *α* = 0.05.

## 3. Ethical Approval

All subjects gave their informed consent for inclusion before they participated in the study. The study protocol was approved by the Ethics Committee of Śląska Izba Lekarska in Katowice (project identification code: 45/2015).

## 4. Results and Discussion

### 4.1. Characteristics of the Studied Population

The study group consisted of 26 patients, including 5 women and 21 men, aged 21 to 82 years (47.46 ± 14.49). The control group consisted of 26 individuals, including 19 women and 7 men, aged 13 to 82 years (33.04 ± 16.75). The average age of patients in the study group was higher than in the control group, and the difference was statistically significant (*p*=0.0017). Data on the age of patients are shown in Table 1.

In the present study, men constituted a majority in the study group (80.77%). The relationship between sex and the occurrence of purulent odontogenic inflammations in the soft tissues of the head and neck area is highly statistically significant (*p*=0.0001). The results are presented in Table 2.

**Figure 2:**
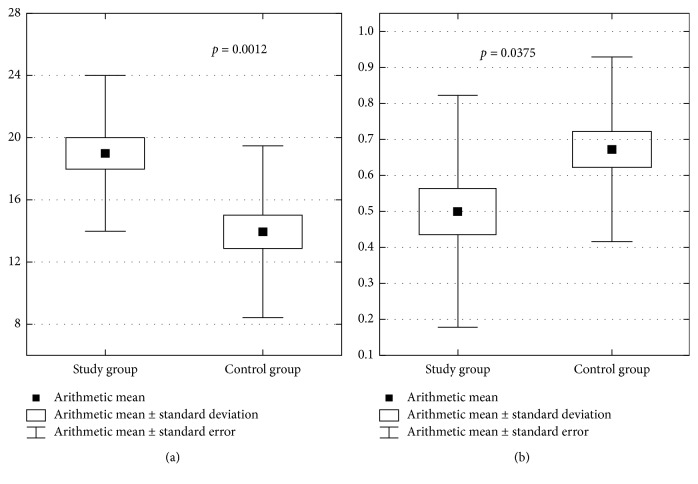
(a) DMF Index values in both groups of patients and (b) Treatment Index values in both groups of patients.

The DMF Index was 18.96 ± 4.94 for patients in the study group and 13.96 ± 5.52 for those in the control group. Lower values of the DMF Index were found in patients in the control group. The differences were statistically significant (*p*=0.0012). Treatment Index values for patients in the study and control groups were 0.50 ± 0.32 and 0.67 ± 0.26, respectively. Higher values of the Treatment Index were found in patients in the control group. The differences were statistically significant (*p*=0.0375). The results are shown in Figure 2.

**Figure 3:**
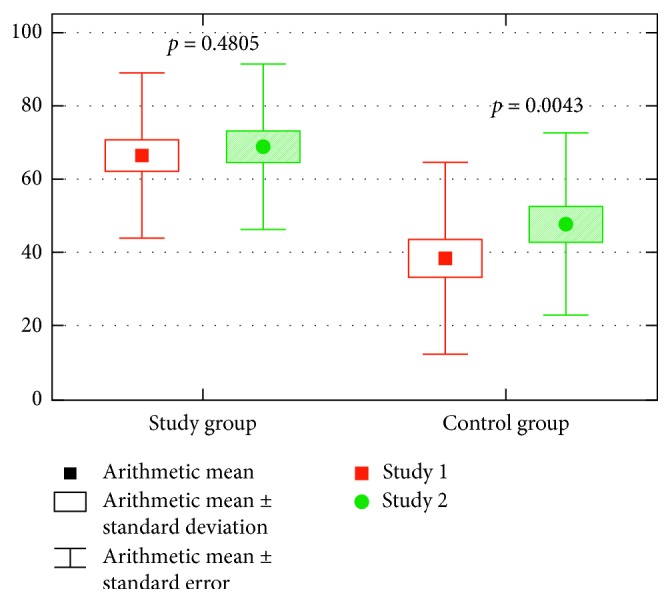
The API values in both groups of patients in study I and study II.

The API values in study I were 66.50 ± 22.08 for patients in the study group and 38.58 ± 26.11 for patients in the control group, whereas in study II they were 68.88 ± 22.28 and 47.85 ± 24.82, respectively. The API value was lower in the control group than in the study group. The difference was statistically significant, both in study I (*p*=0.0001) and in study II (*p*=0.0023). There was also a statistically significant increase in the API in the control group between study I and study II (*p*=0.0043). The data are presented in Figure 3.

### 4.2. Drug Susceptibility Assessment

A total of 67 strains from 31 species of potentially pathogenic microorganisms from the material collected from the tested subjects were assessed. The drugs studied included: penicillin (P), amoxicillin with clavulanic acid (AUG), vancomycin (Va), piperacillin with tazobactam (TZP), clindamycin (CC), metronidazole (MZ), gentamicin (G), biseptol (Bs), cefuroxime (CXM), ciprofloxacin (CIP), cefepime (FEP), and ampicillin (AM). Patients participating in the study were given amoxicillin with clavulanic acid (22 patients) or clindamycin (19 patients). No indications for antibiotic therapy were found in 11 patients. When assessing the sensitivity of bacteria according to the EUCAST guidelines, 100% sensitivity to the tested antibiotics was found in the case of the following drugs:

- In the study group: gentamicin, cefuroxime, and ciprofloxacin
- In the control group: gentamicin and sulfamethoxazole with trimethoprim (biseptol) and cefepime

Comparing the sensitivity to drugs used in patients in this study, the percentage of bacterial strains sensitive to amoxicillin and clavulanic acid in the study and control groups was 78.13% and 81.48% (*p*=0.75), respectively, and the percentage of bacterial strains sensitive to clindamycin was 96.43% and 80.00% (*p*=0.17). The studied bacteria were found to be least sensitive to ampicillin, resulting in a total lack of sensitivity in the case of the study group and 14.29% of sensitive strains in the case of the control group. Among Gram-positive anaerobic bacteria, the highest percentage of susceptible strains was found in the case of amoxicillin with clavulanic acid and metronidazole (100.00%), and clindamycin was found to be effective in 78.57% of strains. In the case of Gram-negative anaerobic bacteria, the highest percentage of susceptible strains was also found in the case of amoxicillin with clavulanic acid, followed by clindamycin (98.32% of susceptible strains). According to the EUCAST 8.1 guidelines, in the case of infections caused by anaerobic bacteria, gentamicin, biseptol, cefuroxime, ciprofloxacin, cefepime, and ampicillin are not recommended. In the case of Gram-positive aerobic bacteria, the only drugs recommended for all cultured strains are clindamycin and sulfamethoxazole with trimethoprim. 88.89% of strains were found to be susceptible to clindamycin and 66.67% to sulfamethoxazole with trimethoprim. Amoxicillin with clavulanic acid is not recommended for infections with bacteria from this group. Among Gram-negative aerobic bacteria, gentamicin and ciprofloxacin were those medications that affected all cultured species, for which 100.00% of strains were found to be susceptible, as well as amoxicillin with clavulanic acid (16.67% of susceptible strains). The remaining drugs recommended for bacterial infections of this group included biseptol (95.00% of susceptible strains), cefuroxime (90% of susceptible strains), cefepime (80% of susceptible strains), and ampicillin (3.33% of susceptible strains). According to the EUCAST guidelines, in the case of aerobic infection, the use of natural penicillin, vancomycin, piperacillin with tazobactam, clindamycin, or metronidazole is not recommended. The obtained results of the susceptibility of microorganisms were divided depending on the group (the study group vs. the control group) and are presented in Table 3. Detailed results of the susceptibility of cultured microorganisms divided into four groups (Gram-positive aerobic, Gram-positive anaerobic, Gram-negative aerobic, and Gram-negative anaerobic) are presented in Table 4.

### 4.3. Assessment of Healing

In the study group, 21 patients showed no complications, and on the follow-up visit on the 10^th^ postoperative day, improvement in general and local condition was noted. Abnormal wound healing which required further procedures was found in 5 patients during the follow-up visit:

- 1 patient reported severe pain lasting a week after the extraction of the causative tooth. Finally, after 2 weeks, the pain subsided. The patient did not take antibiotics.
- 3 patients did not report improvement after the purulent lesion incision. Another incision and drainage of purulent content reservoirs at the follow-up visit were necessary. Two of them were taking antibiotics—AUG. One patient did not take antibiotics.
- 1 patient suffered a prolonged outflow of purulent exudate and a trismus persisting for 5 days. Seton was removed after 9 days and the treatment was successful. The patient was taking CC antibiotic.

In 81% (21) of patients in this group, palpable submandibular and/or submental painful nodes were found during the physical examination on the first visit. In the control examination after 10 days, the lymph nodes were palpable and painless in 92% of patients (24 out of 26). In the control group, 24 of 26 patients underwent no complications and the healing period was uneventful:

- 1 patient was diagnosed with dry alveolus on the follow-up visit after 3 days. The patient visits the clinic the next few days to rinse the alveolus with NaCl physiological solution and to apply the acetylsalicylic acid tablet (Nipas). The patient came to the clinic for the last time 10 days after the surgery, revealing a significant improvement in the local condition. The patient was taking an antibiotic—AUG.
- 1 patient reported severe pain and trismus during the follow-up visit 10 days after the procedure. The patient had control visits after 4 weeks and after 3 months, each time reporting persistent pain, which was gradually reduced. It was only after 6 months that the symptoms completely subsided. The patient was taking an antibiotic—AUG.

Comparing the percentage of specific strains to the occurrence of postoperative complications, it was shown that there is a statistically significant relationship between the incidence of complications and the occurrence of strains of Gram-negative aerobic bacteria (*p*=0.0261) (Table 5). 5 patients had Gram-negative aerobic strains and postoperative complications: 3 of them were in the study group and 2 in the control group. All patients were undergoing antibiotic therapy: four of them—AUG and one (in the study group)—CC. The cultured species included: *Enterobacter kobei* (1 strain), *Enterobacter cloacae* (2 strains), *Providencia rustigianii* (1 strain), and *Chryseobacterium indologenes* (1 strain). 4 of the 5 strains strained were AUG resistant, and one (*Chryseobacterium indologenes*) was susceptible. The susceptibility of strains to CC was not determined in any of the studied cases (according to EUCAST 8.1, it is not recommended for these cases).

## 5. Discussion

Literature data show that the microorganisms that cause odontogenic infections include both aerobic and anaerobic bacteria, as well as both Gram-positive and Gram-negative. There is no antibacterial drug that would have such a wide spectrum of activity to effectively counteract all isolated species [18]. The key to success in the empirical antibiotic therapy is to learn which bacterial species are the most common cause of this type of infection, as well as which antibacterial drugs will have the greatest chance of success. In dentistry, the most commonly used antibacterial agents include *β*-lactam antibiotics (penicillins and cephalosporins), lincosamides (clindamycin), macrolides (azithromycin), fluoroquinolones (ciprofloxacin), and nitroimidazole derivatives (metronidazole) [19–21]. A common characteristic of penicillins and cephalosporin is the *β*-lactam ring, which, by combining with the penicillin-binding protein (PBP), is responsible for the bactericidal activity of the antibiotic [22]. Natural penicillins, sensitive to *β*-lactamases, are characterized by a narrow spectrum of antibacterial activity, mainly directed against Gram-positive bacteria. Penicillins with an extended spectrum of activity exhibit a much wider spectrum of antibacterial activity. They act on all microorganisms susceptible to natural penicillin, as well as a number of other pathogens responsible for the development of paramaxillary infections, including *Haemophilus influenzae*, *Haemophilus parainfluenzae*, *Escherichia coli*, and *Proteus mirabilis*. However, there are still many strains resistant to their activity, including those belonging to *Enterobacter*, *Citrobacter*, *Klebsiella*, or *Pseudomonas aeruginosa* species [23]. Certain *β*-lactamases induced upon the growth of multidrug-resistant (MDR) strains with antibiotics are important in conferring resistance to antibiotics [24]. At this point, *β*-lactamase inhibitors, e.g., clavulanic acid, should be mentioned. Their use allows the deactivation of the majority of *β*-lactamases produced by Gram-negative bacteria, including *Enterobacter* spp., *Klebsiella pneumoniae*, or *Pseudomonas aeruginosa* mentioned above. The combination of the already effective amoxicillin with the *β*-lactamase inhibitor creates a mixture with a huge spectrum of antibacterial activity and a broad therapeutic potential [25]. Cephalosporins are similar in terms of their activity. Oral cephalosporin III, available in the oral form (e.g., cefuroxime axetil), is widely used in outpatient dental surgery, demonstrating effects on bacteria that often cause odontogenic infections, including *Streptococcus* spp., *Staphylococcus* spp. (except MRSA), and *Haemophilus influenzae* (also for strains resistant to penicillin) [26, 27]. In addition, they have good permeability to bone tissue and relatively high resistance to *β*-lactamases [28]. Another type of activity is demonstrated by the widely used lincomycin derivative, clindamycin. By connecting to the 50s ribosomal subunit of a bacterial cell, it inhibits the elongation of the polypeptide chain, which is the basis of its bacteriostatic activity [29]. This drug is effective against bacteria that cause odontogenic inflammation, including *Staphylococcus* spp. (also MRSA), *Streptococcus* spp., *Prevotella melaninogenica*, *Fusobacterium* spp., *Mycoplasma pneumoniae*, and *Clostridium perfringens*. It is also characterized by excellent penetration into bone tissue and hard dental tissues [30]. Clindamycin is not effective in the case of i.a. *Pseudomonas aeruginosa*, and it is also not very potent in infections caused by Gram-negative aerobic bacteria; hence, it is recommended to combine it with third-generation cephalosporins (e.g. cefuroxime). This combination provides a broad spectrum of activity against the majority of Gram-positive and Gram-negative bacteria, both aerobic and anaerobic [25]. Unfortunately, this antibiotic induces a strong dysbacteriosis of the gastrointestinal tract, which in 10–20% of patients may be the cause of persistent diarrhoea, and in combination with the presence of the *Clostridium difficile* strain in the intestines, it is responsible for the occurrence of pseudomembranous colitis [31, 32]. Among the derivatives of nitroimidazole, metronidazole is often used as a bactericide by blocking the synthesis of DNA within a bacterial cell. It is a drug that works particularly well in anaerobic conditions—anaerobic and relatively aerobic bacterial environment, as well as anaerobic protozoa. Bacteria showing a high degree of sensitivity to metronidazole include *Veillonella* spp., *Fusobacterium* spp., *Prevotella* spp., *Peptococcus* spp., *Clostridium* spp., and above all the *Clostridium difficile* species. It should not be combined with bacteriostatic clindamycin. Its combination with amoxicillin or cefuroxime is common and effective [25]. Mücke et al. [14] examined 205 patients diagnosed with perimandibular abscesses and divided them into two groups. The first one was subjected to intraoral incision of the lesion under local anaesthesia, immediately after the patient reported the symptoms. In the case of the remaining patients, the lesions were incised extraorally in general anaesthesia. The necessity to prepare the procedure, including anaesthetic consultation, in each case delayed the implementation of the treatment. In the first group, it was more often necessary to perform repeated surgical procedures (including a second, extraoral incision). However, in these patients, better wound healing effects were observed, together with fewer inflammatory complications (*p* < 0.00001), and the average duration of hospital stay was shorter than in the second group (*p*=0.049). There was also a positive correlation between the healing effects and the use of amoxicillin with clavulanic acid, which the authors recommend as a first-line drug in the case of a perimandibular abscess. This study proves that in the case of purulent inflammations in the head and neck area, the key element of treatment is the elimination of their source (removal of the causative tooth), as well as the drainage of purulent content (incision). The most important factor affecting the outcome of the treatment is its fast implementation [14]. Orzechowska et al. [33], when analysing the bacterial flora present in odontogenic inflammatory changes, noted a significant predominance of Gram-positive bacteria (74.5%) in comparison with Gram-negative (24.4%) microorganisms. The most common bacteria were *Streptococcus mitis* and *Streptococcus oralis*. Significant immunisation of Gram-positive organisms tested for all antibacterial agents over the period of 5 years was noticed. The highest increase in microbial resistance was observed in the case of ampicillin and imipenem [33]. In the present study, significant resistance of the cultured bacteria to ampicillin was also found (only one strain of *Klebsiella oxytoca* was found to be susceptible in all examined cases, which is 7.14% of the tested bacteria). All the strains, however, turned out to be susceptible to imipenem. Similar studies conducted by Sobottka et al. [34] showed that 98% of strains cultured from odontogenic inflammatory changes appeared to be susceptible to moxifloxacin and 96% of strains to amoxicillin/clavulanic acid. Clindamycin was effective in 60% of the studied microorganisms [34]. Rams et al. [35] were investigating the sensitivity of bacterial flora in chronic periodontitis and reported the presence of drug-resistant strains in 74.2% of studied patients, among whom 55.0% had strains resistant to doxycycline, 43.3% of patients had strains resistant to amoxicillin, and 26.5% of patients had clindamycin-resistant strains [35]. A lot of research has been conducted to explain the desirability of prophylactic antibiotic therapy in healthy patients before and/or after ITM removal [36]. The positive effect of the drug used on postoperative healing is proven [36–43]. At the same time, many authors show a lack of legitimacy of prophylactic antibiotic therapy, citing a number of negative effects of its abuse [44–47]. Gbotolorun et al. [45] were investigating the group of patients receiving amoxicillin and metronidazole after tooth extraction and found the presence of inflammatory complications in 16% of individuals, compared to 12% in the placebo control group [45]. Xue et al. [44] examined the quality of wound healing after the removed ITM depending on the perioperative antibiotic use. The study was conducted on 207 patients, each of them had a total of 2 ITM removed during 2 visits. In all cases, one treatment was carried out with the use of an antibiotic (amoxicillin or clindamycin, from 1 hour before the surgery to 3 days after the procedure). In the second group, placebo was used instead of an antibiotic. There were no statistically significant differences in postoperative wound healing, neither did any inflammatory complications occur [44]. In contrast to previous investigators, López-Cedrún et al. [48] showed that the use of antibiotic (amoxicillin) significantly affected the postoperative pain and the incidence of complications, i.e., postoperative wound infection, trismus, fever, or dysphagia. In addition, it was shown that the best effects in preventing complications after the removal of ITM were obtained by using a postoperative antibiotic [48]. Schüssl et al. [30] examined the concentration of antibiotics (amoxicillin and clindamycin) in dental hard tissues after oral administration for 60–120 minutes before extraction. The observed concentration of antibiotics exceeded the MIC90 value for some potentially pathogenic microorganisms present in the oral cavity, which confirms the validity of using these drugs, especially in the case of heavier and more vulnerable ITM removal procedures [30]. The abuse of antibiotics in dentistry is a problem known all over the world [49, 50]. This phenomenon is strictly related to the formation of multidrug-resistant strains of bacteria and causes complications in many different branches of medicine [12, 24, 51]. Marra et al. [20] showed that although in the years 1996–2013 the total frequency of prescribing antibiotics by physicians fell by 12.77%, at the same time the frequency of prescribing antibiotics by dentists increased by 62.2% [20]. In the Czech Republic, the frequency of prescribing amoxicillin and clindamycin increased by 60% in the years 2006–2012 [52]. In Germany, amoxicillin and clindamycin are also the most frequently prescribed antibiotics by dentists. In 2015, they were prescribed in 45.8% and 31.7% of all cases, respectively [53]. Also in Poland, the number of administered antibiotics is constantly increasing. Detailed studies carried out by Chlabicz et al. [54] show that in 2004–2008, over 50% of patients treated with antibiotics used penicillins, in particular amoxicillin, alone or with the addition of *β*-lactamase inhibitors [54]. The probable causes of the abuse of antibiotics by dentists are frequent errors in the treatment of odontogenic inflammation (antibiotic therapy instead of causative treatment), but also the slow adaptation to the latest recommendations, limiting the use of antibiotics in patients with cardiac defects, population aging, or popularisation of dental implants and related complications [35, 50, 55]. To sum up, the most important aspect of an effective treatment of odontogenic inflammation involves the correct diagnosis and immediate surgical intervention, with the antibiotic aspect being of secondary importance [56]. The broad spectrum of activity and the relatively low risk of side effects favour the use of penicillin. The benefits of using clindamycin are associated with its excellent penetration of bone tissue, which is the focus of odontogenic inflammation. Unfortunately, often the only action taken by dentists in cases of the development of acute inflammation is antibiotic therapy without the implementation of a surgical procedure. This is inconsistent with the modern medical knowledge and exposes the patient to a number of serious potential complications [57]. Treatment should be preceded by a thorough medical interview, and it should be tailored individually to each patient. Numerous evidence points to the low effectiveness of prophylactic therapy in healthy people [45, 47]. It should be considered whether the potential benefits outweigh the risk of adverse effects [58]. The conclusions based on the results of the planned testing can be practically used during the updating and possible modification of the recommendations regarding empiric antibiotic treatment used both in patients with acute oral inflammatory conditions and in patients after elective surgery in an outpatient procedure.

## 6. Conclusions

1. Differences in susceptibility of cultured bacterial flora were found, depending on the type of the bacteria. Among the anaerobic bacteria, the highest percentage of susceptible strains was found for amoxicillin with clavulanic acid and clindamycin. Among the aerobic bacteria, the highest number of bacterial strains was found to be susceptible to gentamicin, ciprofloxacin, and cefuroxime.
2. In cases of odontogenic inflammation, the primary treatment should be implementation of an appropriate surgical procedure. In the presence of systemic symptoms, it seems reasonable to use an additional combination of antibiotics (amoxicillin with cefuroxime or ciprofloxacin or clindamycin with cefuroxime) to provide a broad spectrum of antibacterial activity.
3. The procedure of removal of the third impacted molar leads to a decrease in oral hygiene during the first week after its implementation, which was proven by a statistically significant increase in API tested immediately before and a week after surgery.

## Figures and Tables

**Table 1:** Age of patients in the study and control groups.

	Study group (mean ± SD)	Control group (mean ± SD)	*p*
Age of patients (years)	47.5 ± 14.5	33.0 ± 16.8	0.0017

**Table 2:** Sex of patients in the study and control groups.

	Study group	Control group	Total
Women	5	19	24
Percentage (%)	19.23	73.08	46.15

Men	21	7	28
Percentage (%)	80.77	26.92	53.85

Total	26	26	52

**Table 3:** Comparison of microbial susceptibility to antibacterial drugs in the study and control groups.

Antibacterial agent		Control group	Study group	*p*
P	*N*	27	19	*p*=0.6113
	Ns	19	12	
	**%**	**70.37**	**63.16**	

AUG	*N*	32	27	*p*=0.7517
	Ns	25	22	
	**%**	**78.13**	**81.48**	

Va	*N*	25	19	*p*=0.5375
	Ns	18	12	
	**%**	**72.00**	**63.16**	

TZP	*N*	21	19	*p*=0.5593
	Ns	17	16	
	**%**	**80.95**	**84.21**	

CC	*N*	28	20	*p*=0.1746
	Ns	27	16	
	**%**	**96.45**	**80.00**	

MZ	*N*	25	17	*p*=0.1204
	Ns	10	11	
	**%**	**40.00**	**64.71**	

G	*N*	7	9	*p*=1.0000
	Ns	7	9	
	**%**	**100**	**100.00**	

Bs	*N*	10	9	*p*=0.1238
	Ns	7	9	
	**%**	**70.00**	**100.00**	

CXM	*N*	7	7	*p*=0.5000
	Ns	7	6	
	**%**	**100.00**	**85.71**	

CIP	*N*	7	9	*p*=0.5625
	Ns	7	8	
	**%**	**100.00**	**88.89**	

FEP	*N*	7	7	*p*=0.0962
	Ns	4	7	
	**%**	**57.14**	**100.00**	

AM	*N*	7	7	*p*=0.5000
	Ns	0	1	
	**%**	**0.00**	**14.29**	

P, penicillin; AUG, amoxicillin with clavulanic acid; Va, vancomycin; TZP, piperacillin with tazobactam; CC, clindamycin; MZ, metronidazole; G, gentamicin; Bs, sulfamethoxazole with trimethoprim (biseptol); CXM, cefuroxime; CIP, ciprofloxacin; FEP, cefepime; AM, ampicillin.

**Table 4:** Distribution of susceptibility in individual groups of bacteria depending on the antibacterial drug.

		Antibacterial agent																																			
		P			AUG			Va			TZP			CC			MZ			G			Bs			CXM			CIP			FEP			AM		
Species	*N*	*n*	Ns	%	*n*	Ns	%	*n*	Ns	%	*n*	Ns	%	*n*	Ns	%	*n*	Ns	%	*n*	Ns	%	*n*	Ns	%	*n*	Ns	%	*n*	Ns	%	*n*	Ns	%	*n*	Ns	%
Gram-positive anaerobic																																					
* Actinomyces naeslundii*	17	17	12	70.59	17	17	100.00	17	11	64.71	17	15	88.24	17	15	88.24	17	13	76.47	0	0	NA	0	0	NA	0	0	NA	0	0	NA	0	0	NA	0	0	NA
* Actinomyces odontolyticus*	4	4	3	75.00	4	4	100.00	4	3	75.00	4	4	100.00	4	4	100.00	4	0	0.00	0	0	NA	0	0	NA	0	0	NA	0	0	NA	0	0	NA	0	0	NA
* Propionibacterium propionicum*	4	4	2	50.00	4	4	100.00	4	3	75.00	2	1	50.00	4	4	100.00	4	0	0.00	0	0	NA	0	0	NA	0	0	NA	0	0	NA	0	0	NA	0	0	NA
* Clostridium perfringens*	3	3	3	100.00	3	3	100.00	3	3	100.00	3	3	100.00	3	3	100.00	3	0	0.00	0	0	NA	0	0	NA	0	0	NA	0	0	NA	0	0	NA	0	0	NA
* Actinomyces israelii*	3	3	2	66.67	3	3	100.00	3	2	66.67	2	1	50.00	2	2	100.00	2	0	0.00	0	0	NA	0	0	NA	0	0	NA	0	0	NA	0	0	NA	0	0	NA
* Clostridium sporogenes*	2	2	1	50.00	2	2	100.00	2	0	0.00	2	0	0.00	2	1	50.00	2	2	100.00	0	0	NA	0	0	NA	0	0	NA	0	0	NA	0	0	NA	0	0	NA
* Actinomyces meyeri*	2	2	2	100.00	2	2	100.00	2	2	100.00	2	2	100.00	2	2	100.00	2	0	0.00	0	0	NA	0	0	NA	0	0	NA	0	0	NA	0	0	NA	0	0	NA
* Clostridium novyi biovar A*	2	2	2	100.00	2	2	100.00	2	2	100.00	2	2	100.00	2	1	50.00	2	2	100.00	0	0	NA	0	0	NA	0	0	NA	0	0	NA	0	0	NA	0	0	NA
* Clostridium butyricum*	1	1	0	0.00	1	1	100.00	1	0	0.00	1	1	100.00	1	1	100.00	1	1	100.00	0	0	NA	0	0	NA	0	0	NA	0	0	NA	0	0	NA	0	0	NA
* Clostridium chauvoei*	1	1	0	0.00	1	1	100.00	1	0	0.00	1	0	0.00	1	1	100.00	1	1	100.00	0	0	NA	0	0	NA	0	0	NA	0	0	NA	0	0	NA	0	0	NA
* Clostridium novyi*	1	1	0	0.00	1	1	100.00	1	0	0.00	1	1	100.00	1	1	100.00	1	1	100.00	0	0	NA	0	0	NA	0	0	NA	0	0	NA	0	0	NA	0	0	NA
* Clostridium tertium*	1	1	0	0.00	1	1	100.00	1	1	100.00	1	1	100.00	1	1	100.00	1	1	100.00	0	0	NA	0	0	NA	0	0	NA	0	0	NA	0	0	NA	0	0	NA
* Actinomyces viscosus*	1	1	1	100.00	1	1	100.00	1	1	100.00	0	0	NA	1	1	100.00	1	0	0.00	0	0	NA	0	0	NA	0	0	NA	0	0	NA	0	0	NA	0	0	NA

Gram-negative anaerobic																																					
* Bacteroides ovatus*	1	1	1	100.00	1	1	100.00	1	1	100.00	1	1	100.00	0	0	NA	0	0	NA	0	0	NA	0	0	NA	0	0	NA	0	0	NA	0	0	NA	0	0	NA
* Fusobacterium nucleatum*	1	1	1	100.00	1	1	100.00	1	1	100.00	1	1	100.00	1	1	100.00	1	0	0.00	0	0	NA	0	0	NA	0	0	NA	0	0	NA	0	0	NA	0	0	NA

Gram-positive aerobic																																					
* Streptococcus pneumoniae*	3	1	1	100.00	0	0	NA	0	0	NA	0	0	NA	3	2	66.67	0	0	NA	0	0	NA	2	0	0.00	0	0	NA	1	0	0.00	0	0	NA	0	0	NA
* Staphylococcus aureus*	2	0	0	NA	0	0	NA	0	0	NA	0	0	NA	2	2	100.00	0	0	NA	1	1	100.00	2	2	100.00	0	0	NA	0	0	NA	0	0	NA	0	0	NA
* Staphylococcus epidermidis*	1	1	0	0.00	0	0	NA	0	0	NA	0	0	NA	1	1	100.00	0	0	NA	0	0	NA	1	1	100.00	0	0	NA	0	0	NA	0	0	NA	0	0	NA

Gram-negative aerobic																																					
* Klebsiella oxytoca*	3	0	0	NA	3	1	33.33	0	0	NA	0	0	NA	0	0	NA	0	0	NA	2	2	100.00	3	3	100.00	3	3	100.00	3	3	100.00	3	3	100.00	3	1	33.33
* Enterobacter cloacae*	2	0	0	NA	2	0	0.00	0	0	NA	0	0	NA	0	0	NA	0	0	NA	2	2	100.00	2	2	100.00	2	2	100.00	2	2	100.00	2	0	0.00	2	0	0.00
* Escherichia coli*	2	0	0	NA	1	0	0.00	0	0	NA	0	0	NA	0	0	NA	0	0	NA	1	1	100.00	1	1	100.00	1	1	100.00	1	1	100.00	1	1	100.00	1	0	0.00
* Klebsiella pneumoniae*	2	0	0	NA	2	1	50.00	0	0	NA	0	0	NA	0	0	NA	0	0	NA	2	2	100.00	2	1	50.00	2	2	100.00	2	2	100.00	2	2	100.00	2	0	0.00
* Burkholderia cepacia*	1	0	0	NA	1	0	0.00	0	0	NA	0	0	NA	0	0	NA	0	0	NA	1	1	100.00	1	1	100.00	0	0	NA	1	1	100.00	1	1	100.00	0	0	NA
* Chryseobacterium indologenes*	1	0	0	NA	1	1	100.00	0	0	NA	0	0	NA	0	0	NA	0	0	NA	1	1	100.00	0	0	NA	1	1	100.00	1	1	100.00	1	1	100.00	1	0	0.00
* Enterobacter aerogenes*	1	0	0	NA	1	0	0.00	0	0	NA	0	0	NA	0	0	NA	0	0	NA	1	1	100.00	1	1	100.00	1	1	100.00	1	1	100.00	1	1	100.00	1	0	0.00
* Enterobacter kobei*	1	0	0	NA	1	0	0.00	0	0	NA	0	0	NA	0	0	NA	0	0	NA	1	1	100.00	1	1	100.00	1	1	100.00	1	1	100.00	1	1	100.00	1	0	0.00
* Hafnia alvei*	1	0	0	NA	1	0	0.00	0	0	NA	0	0	NA	0	0	NA	0	0	NA	1	1	100.00	1	1	100.00	1	1	100.00	1	1	100.00	1	0	0.00	1	0	0.00
* Providencia rustigianii*	1	0	0	NA	1	0	0.00	0	0	NA	0	0	NA	0	0	NA	0	0	NA	1	1	100.00	1	1	100.00	1	0	0.00	1	1	100.00	0	0	NA	1	0	0.00
* Serratia odorifera*	1	0	0	NA	1	0	0.00	0	0	NA	0	0	NA	0	0	NA	0	0	NA	1	1	100.00	1	1	100.00	1	1	100.00	1	11	100.00	1	1	100.00	1	0	0.00

*N*, number of strains of a given microorganism cultured; *n*, number of strains for which susceptibility to a given antibiotic has been determined; Ns, number of strains shown to be susceptible to a given antibacterial agent; %, percentage of bacteria susceptible to a given antibacterial agent; NA, no indications to assess the effect of a particular antibacterial agent on the test microorganism (according to the EUCAST 8.1 guidelines); P, penicillin; AUG, amoxicillin with clavulanic acid; Va, vancomycin; TZP, piperacillin with tazobactam; CC, clindamycin; MZ, metronidazole; G, gentamicin; Bs, sulfamethoxazole with trimethoprim (biseptol); CXM, cefuroxime; CIP, ciprofloxacin; FEP, cefepime; AM, ampicillin.

**Table 5:** Comparison of the percentage of individual groups of bacteria to the occurrence of complications in the postoperative period.

Bacteria	Total number of patients (*n* = 52)	Postoperative complications		*p*
		Not present (*n* = 45)	Present (*n* = 7)	
Gram-positive anaerobic	48.97% (25/52)	46.67% (21/45)	57.14% (4/7)	0.9128
Gram-negative anaerobic	3.85% (2/52)	4.44% (2/45)	0.00% (0/7)	0.6259
Gram-positive aerobic	11.54% (6/52)	13.33% (6/45)	0.00% (0/7)	0.6956
Gram-negative aerobic	28.85% (15/52)	22.22% (10/45)	71.43% (5/7)	0.0261
Gram-positive anaerobic vs. Gram-negative anaerobic, *p*		<0.0001	0.0350	—
Gram-positive aerobic vs. Gram-negative aerobic, *p*		0.2728	0.0105	—
Gram-positive anaerobic vs. Gram-positive aerobic, *p*		0.0006	0.0350	—
Gram-negative anaerobic vs. Gram-negative aerobic, *p*		0.0136	0.0105	—

## Data Availability

The data used to support the findings of this study are available from the corresponding author upon request.
